# Associations of fat mass and muscle function but not lean mass with cognitive impairment: The Yishun Study

**DOI:** 10.1371/journal.pone.0256702

**Published:** 2021-08-26

**Authors:** Nien Xiang Tou, Shiou-Liang Wee, Benedict Wei Jun Pang, Lay Khoon Lau, Khalid Abdul Jabbar, Wei Ting Seah, Kenneth Kexun Chen, Tze Pin Ng

**Affiliations:** 1 Geriatric Education and Research Institute, Singapore, Singapore; 2 Health and Social Sciences Cluster, Singapore Institute of Technology, Singapore, Singapore; 3 Program of Health Services and System Research, Duke-National University of Singapore Medical School, Singapore, Singapore; 4 Department of Psychological Medicine, National University of Singapore, Singapore, Singapore; University of New South Wales, AUSTRALIA

## Abstract

**Background:**

Sarcopenia and obesity are reportedly associated with risk of cognitive decline, and sarcopenic obesity (SO) heightens the risk, but the evidence is sparse and inconclusive. This study aimed to examine the association between SO and cognitive impairment.

**Methods:**

A total of 542 community-dwelling adults aged between 21 and 90 years were recruited. All participants were assessed for body composition (dual X-ray absorptiometry), handgrip strength (HGS), gait speed (GS), and cognitive function (Repeatable Battery for the Assessment of Neuropsychological Status). Sarcopenia was defined by the presence of low appendicular lean mass index (ALMI) and low HGS or low GS according to the 2019 Asian Working Group for Sarcopenia criteria, and obesity was defined based on the upper two quintiles of fat mass index (FMI).

**Results:**

Sarcopenia alone or in combination with obesity were not significantly associated with cognitive impairment after controlling for confounding variables. Obesity on its own was significantly associated with greater odds of impaired attention (OR: 2.05, 95%CI 1.12–3.82). Low ALMI was not associated, but low HGS, slow GS, and high FMI were individually associated with cognitive impairment: low HGS and immediate memory (OR: 1.91, 95% CI 1.04–3.49); low GS and immediate memory (OR: 2.17, 95% CI 1.26–3.72); high FMI and attention (OR: 2.06, 95% CI 1.22–3.51). Co-occurring high FMI with either low HGS or slow GS exacerbated the observed odds of global and domain-specific (attention, visuospatial) cognitive impairment.

**Conclusions:**

Lean mass is not relevant, whereas muscle strength and physical performance or adiposity are relevant in defining sarcopenia or sarcopenic obesity in terms of their cognitive impacts.

## Introduction

Around 50 million people live with dementia worldwide, and this number is projected to increase to 152 million by 2050 [1]. While there is currently no cure for dementia, there is good evidence in support of the prevention or delaying dementia in at-risk individuals in the pre-dementia stage [2]. Mild cognitive impairment is a pre-dementia stage that can be identified years before onset of dementia.

Sarcopenia is a geriatric syndrome, characterised by the progressive loss of skeletal muscle mass and muscle strength with age [3]. With its increasing prevalence in Asia [4], sarcopenia is a public health challenge given its association with functional decline and physical disability [5]. It is also associated with a >2-fold increased risk of cognitive impairment [6,7], plausibly attributed to shared pathophysiology between the two conditions [8,9].

Concurrent with reduction in muscle mass, increase in total body fat and abdominal adiposity is another age-associated phenomenon [10]. Studies have reported mixed findings of a positive relationship between obesity and cognitive impairment [11,12], or a negative relationship [13] depending on age and sex of subjects [14–17]. While obesity in mid-life (45-65y) is an established risk factor for future dementia [2], being obese (body mass index (BMI) ≥30) in late-life (>65y) reduced the risk of dementia compared to normal BMI [18]. This “obesity paradox” in dementia risk across time may be explained by the age-related body composition changes in the trajectory toward sarcopenia and disability [18]. The conflicting evidence and paradox are likely due to the use of BMI to measure obesity because BMI does not differentiate between fat and fat-free mass [19]. Furthermore, most studies only assessed global cognition functioning and did not distinguish between different domains of cognition [14,15].

The coexistence of sarcopenia and obesity—sarcopenic obesity (SO) [20], has been purported to have additive exacerbating effects on cognitive performance [9,21]. However, the evidence on this relationship remained inconclusive. Two studies on community-dwelling older adults found that SO was associated with poorer cognitive performance than either sarcopenia or obesity alone [21,22], while another study reported similar results only among older adults aged 70 and above but not among those aged 60–69 years [9].

The lack of an age appropriate and standardised obesity definition poses a challenge in studying the negative health consequences associated with SO [23]. The aforementioned studies have used percentage body fat [21,22], and waist circumference [9] to determine obesity. Recently, we presented data to show that fat mass index (FMI) may be the most optimal criterion for SO among the obesity measures, as it accounts for body size differences and improves the association between adiposity and physical function [24]. To date, no studies have examined the relationship between cognition and SO based on FMI.

Given the global pandemic of obesity and that sarcopenia could also occur in middle-aged adults [25,26], it is paramount to understand the association between SO and cognitive impairment in a general adult population inclusive of all age groups. Hence, this study aimed to examine the association between SO and global as well as domain-specific aspects of cognition among healthy community-dwelling adults. To better understand this association, this study also examined the association between individual components of SO and cognitive impairment.

## Materials and methods

### Participants

This is part of the Yishun Study, a cross-sectional study that determined the normative values and prevalence of sarcopenia among a representative sample of 542 community-dwelling adults aged 21–90 years in Singapore. The sampling method has been detailed elsewhere [25]. The final analysis sample consists of 535 participants (308 women, 227 men) who had complete body composition data. All participants provided written informed consent prior to participation and ethics approval was obtained from the National Healthcare Group Domain Specific Review Board (2017/00212).

### Anthropometric and body composition measurements

Body weight and height were measured using a digital balance and stadiometer (Seca, GmbH & Co. KG, Hamburg, Germany). BMI was calculated as weight (kg) divided by height (m) squared. Bone mineral density, total body fat percentage, fat mass and appendicular lean mass were measured using dual X-ray absorptiometry (DXA; Discovery WI, Hologic, Inc., Marlborough, USA). FMI and appendicular lean mass index (ALMI) were used as measures of fat mass and muscle mass respectively in the present study to account for height-associated differences in body composition [4,27]. Both FMI and ALMI were calculated as fat mass (kg) and appendicular lean mass (kg) divided by height (m) squared, where fat mass equals to total body fat mass and appendicular lean mass equals to the sum of lean mass in the upper and lower limbs.

### Muscle strength and physical function assessment

Handgrip strength (HGS) was used as indication of muscular strength of participants [28] and physical function was measured using usual gait speed (GS) [29]. HGS was assessed using a dynamometer (Jamar Plus+ Dynamometer; Patterson Medical, Evergreen Boulevard, Cedarburg, USA), and the highest of four readings (two trials per arm) recorded. GS was measured using the 6 m GAITRite Walkway (CIR Systems Inc., Sparta. New Jersey, USA) with a 2 m lead in and out phase, and the average speed (three trials) recorded.

### Sarcopenia, obesity and sarcopenic obesity assessment

Sarcopenia was assessed using the latest Asian Working Group for Sarcopenia (AWGS) algorithm [4]. Poor physical function was defined as GS <1.0 m/s, low muscle mass as ALMI <7.0 and <5.4 kg/m2, and low muscle strength as HGS <28 and <18 kg for men and women respectively. Sarcopenia was defined as the presence of low muscle mass and poor muscle strength and/or physical performance [4]. Given that there is presently no consensus on the definition of obesity [30], obesity was defined in this study based on the top two quintiles of FMI. The cut-off points adjusted for gender were ≥ 7.63 kg/m^2^ and ≥ 9.93 kg/m^2^ for men and women respectively. Participants were categorised into four non-overlapping groups based on presence and absence of sarcopenia and obesity: SO (sarcopenic and obese), sarcopenic (sarcopenic and non-obese), obese (non-sarcopenic and obese) and normal (non-sarcopenic and non-obese).

### Cognitive function assessment

Cognitive function was measured using the Repeatable Battery for the Assessment of Neuropsychological Status (RBANS), which encompassed 12 subtests to assess five cognitive domains: immediate memory, visuospatial/constructional abilities, language, attention, and delayed memory [31]. Total scale index and domain-specific index scores for all participants were scored according to the RBANS manual [32], whereby each score was expressed as a standardised score with a mean of 100 and standard deviation of 15. Cognitive impairment was defined using a cut-off index score <80, which corresponds to the 9^th^ percentile [32].

### Other measures

Demographic characteristics such as age, gender, education levels, housing type, smoking history and medical history were collected through a questionnaire. Participants also completed the global physical activity questionnaire to report their typical weekly physical activity levels [33].

### Statistical analysis

Differences in variables of interest among the four groups defined by sarcopenia and obesity status were examined with one-way analysis of variance for continuous variables and chi-squared test for categorical variables. Multivariate logistic regressions were performed to examine the associations between SO and impairment in global cognition as well as different domains of cognitive function. Odds ratios (OR) and 95% confidence intervals (CI) were estimated to compare the sarcopenic, obese and SO groups with the normal group. In order to examine the relationship between individual components of SO (muscle strength, muscle mass, physical function, and obesity) and cognitive impairment, participants were dichotomised based on each component and separate logistic regressions were conducted to determine the odds of impairment in different domains of cognitive function. Models were adjusted for age groups, gender, physical activity levels, medical history, and years of education. Statistical significance level was set at *p* < 0.05 and all analyses were performed using R statistical software, version 3.6.3.

## Results

**Fig 1:**
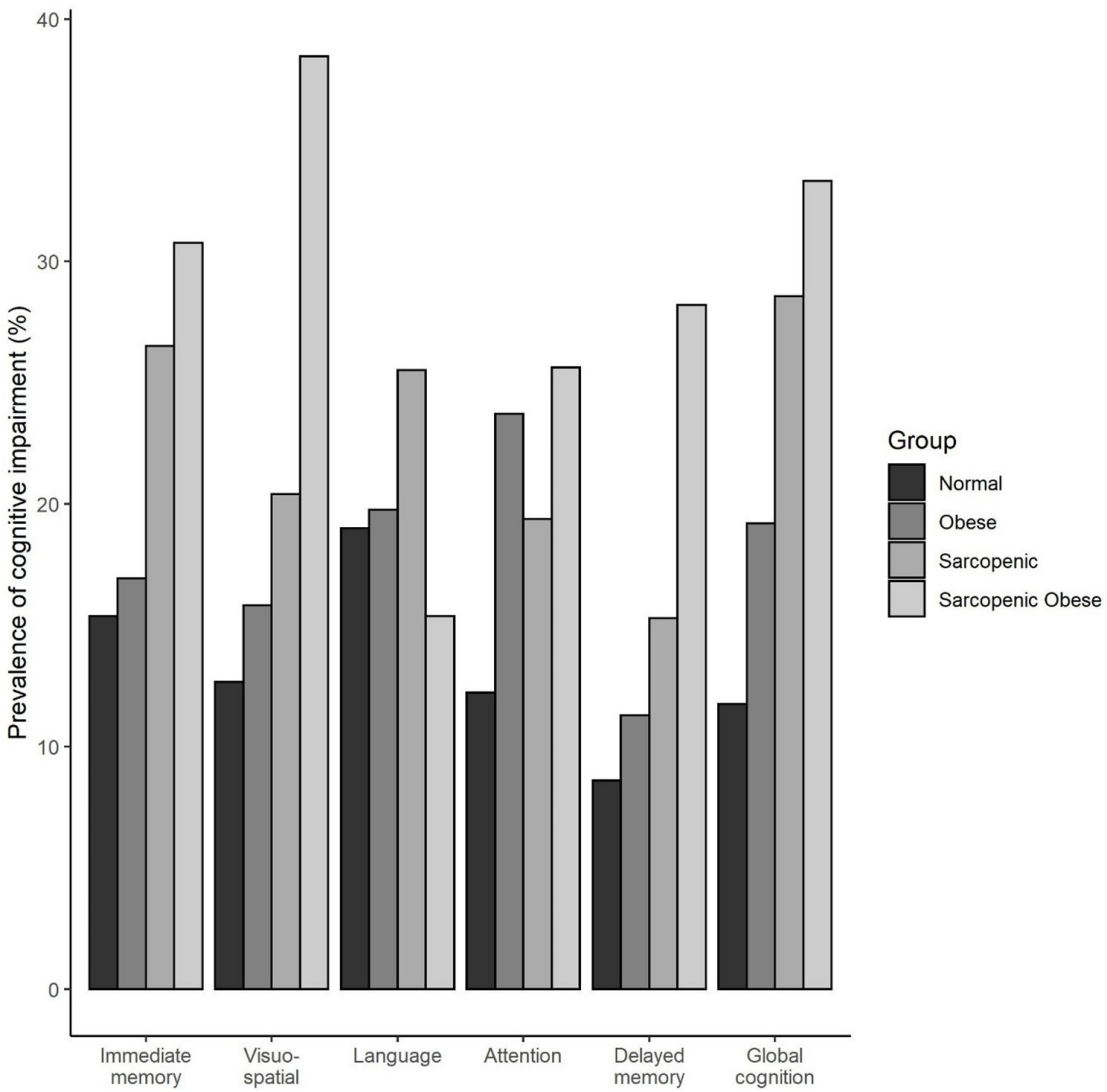
Prevalence of cognitive impairment by sarcopenia and obesity status.

The prevalence of SO, sarcopenia, obesity and normal were 7.3%, 18.3%, 33.1% and 41.3% respectively. Table 1 provides the descriptive characteristics of participants across the four groups. The groups were found to differ in prevalence of medical conditions such as diabetes, hypertension, cardiovascular disease, and high cholesterol. HGS and GS were found to differ significantly between groups, whereby the SO group had the lowest HGS and slowest GS. Both the SO and sarcopenic groups were found to be significantly older and had significantly less education as compared to the obese and normal group. The SO group was also found to be significantly less physically active than the other three groups. The prevalence of cognitive impairment in respective domains across the four groups is shown in Fig 1.

**Table 1:** Descriptive characteristics of participants by sarcopenia and obesity status.

	Sarcopenic Obese (*n* = 39)	Sarcopenic (*n* = 98)	Obese (*n* = 177)	Normal (*n* = 221)	*p*-value
**Age (y)**	75.59 ± 9.00	71.11 ± 14.63	56.12 ± 17.86	51.97 ± 17.95	<0.01*
**Age groups**					<0.01*
21–39 years	0 (0.0%)	6 (6.1%)	41 (23.2%)	64 (29.0%)	
40–59 years	1 (2.6%)	8 (8.2%)	47 (26.6%)	65 (29.4%)	
60–74 years	13 (33.3%)	32 (32.7%)	59 (33.3%)	63 (28.5%)	
≥75 years	25 (64.1%)	52 (53.0%)	30 (16.9%)	29 (13.1%)	
**Female (n, %)**	25 (64.1%)	52 (53.1%)	100 (56.5%)	131 (59.3%)	0.61
**Smoker (n, %)**	2 (5.1%)	8 (8.2%)	20 (11.3%)	29 (13.1%)	0.36
**Education (y)**	6.79 ± 4.93	7.30 ± 5.18	9.95 ± 4.97	10.89 ± 4.55	<0.01*
**PA (MET-min/wk)**	31.69 ± 27.40	49.75 ± 46.98	75.11 ± 105.26	72.74 ± 73.53	<0.01*
**Medical history**					
Diabetes	9 (23.1%)	27 (27.6%)	24 (13.6%)	20 (9.0%)	< 0.01*
Hypertension	29 (74.4%)	49 (50.0%)	65 (36.7%)	53 (24.0%)	< 0.01*
Cardiovascular disease	4 (10.3%)	12 (12.2%)	7 (4.0%)	9 (4.1%)	0.01*
High cholesterol	27 (69.2%)	44 (44.9%)	73 (41.2%)	58 (26.2%)	<0.01*
Stroke	1 (2.6%)	2 (2.0%)	1 (0.6%)	1 (0.5%)	0.36
Depression	2 (5.1%)	1 (1.0%)	4 (2.3%)	1 (0.5%)	0.11
**Body composition**					
BMI (kg/m2)	25.72 ± 1.78	21.48 ± 2.00	29.21 ± 4.43	22.56 ± 2.55	<0.01*
FMI (kg/m2)	10.68 ± 1.58	7.03 ± 1.55	11.42 ± 2.89	7.08 ± 1.72	<0.01*
ALMI (kg/m2)	5.45 ± 0.71	5.35 ± 0.78	7.04 ± 1.33	6.04 ± 1.13	<0.01*
**Physical function**					
HGS (kg)	20.85 ± 6.10	22.27 ± 6.26	30.50 ± 9.96	30.54 ± 8.98	<0.01*
GS (m/s)	0.84 ± 0.15	0.90 ± 0.15	1.07 ± 0.18	1.15 ± 0.17	<0.01*
**Cognitive function**					
RBANS index score	84.82 ± 14.83	86.41 ± 15.41	93.65 ± 17.19	96.28 ± 15.58	<0.01*
Immediate memory domain	84.69 ± 14.14	88.73 ± 16.31	93.77 ± 16.16	95.10 ± 15.64	<0.01*
Visuospatial domain	89.46 ± 19.38	90.13 ± 15.12	95.98 ± 16.88	99.03 ± 16.43	<0.01*
Language domain	89.56 ± 11.84	88.70 ± 15.30	91.20 ± 15.02	91.28 ± 16.12	0.50
Attention domain	88.05 ± 15.10	91.52 ± 15.23	96.11 ± 19.32	100.47 ± 16.95	<0.01*
Delayed memory domain	90.49 ± 17.01	94.41 ± 16.52	98.55 ± 15.89	100.35 ± 14.30	<0.01*

PA, physical activity levels; BMI, body mass index; FMI, fat mass index; ALMI, appendicular lean mass index; HGS, handgrip strength; GS, gait speed; RBANS, Repeatable Battery for the Assessment of Neuropsychological Status.

*p < 0.05.

All data are presented as mean ± SD or number (%).

### Association between cognitive impairment and sarcopenia and obesity status

Table 2 presents the logistic regression analysis results for the association between SO and risk of impairment in different domains of cognitive function. In the unadjusted models, the SO group was found to have significantly higher odds of impairment as compared to the normal group in global cognitive function (OR: 3.75, 95% confidence interval (CI) 1.69–8.13, *p* < .01), as well as domains of immediate memory (OR: 2.44, 95% CI 1.10–5.21, *p* = .023), visuospatial/constructional abilities (OR: 4.31, 95% CI 2.00–9.17, *p* < .01), attention (OR: 2.48, 95% CI 1.05–5.53, *p* = .031), and delayed memory (OR: 4.18, 95% CI 1.76–9.61, *p* < .01). Significant association was also found for the sarcopenic group in the unadjusted models for global cognition (OR: 3.00, 95% CI 1.65–5.49, *p* < .01), and immediate memory (OR: 1.99, 95% CI 1.11–3.54, *p* = .020). However, the risk of cognitive impairment for the SO and sarcopenic groups were not significant in the adjusted models. The obese group was found to have significantly higher odds of association with impaired global cognition (OR: 1.78, 95% CI 1.03–3.13, *p* = .041), and attention (OR: 2.24, 95% CI 1.32–3.84, *p* < .01) in the unadjusted models. After adjusting for confounders, the OR of association between obesity and attention remains significant (OR: 2.08, 95% CI 1.15–3.81, *p* = .021).

**Table 2:** Association between sarcopenic obesity and risk of impairment in different domains of cognitive function.

	Sarcopenic Obese (*n* = 39)		Sarcopenic (*n* = 98)		Obese (*n* = 177)		Normal (*n* = 221)
	OR (95% CI)	*p*	OR (95% CI)	*p*	OR (95% CI)	*p*	
**Global cognitive function**							
model 1	3.75 (1.69–8.13)	< .01**	3.00 (1.65–5.49)	< .01**	1.78 (1.03–3.13)	.041*	1.0
model 2	1.55 (0.62–3.77)	.335	1.46 (0.74–2.89)	.274	1.59 (0.88–2.89)	.125	1.0
model 3	1.62 (0.58–4.47)	.350	1.16 (0.53–2.50)	.706	1.55 (0.80–3.02)	.192	1.0
**Immediate memory**							
model 1	2.44 (1.10–5.21)	.023*	1.99 (1.11–3.54)	.020*	1.12 (0.65–1.92)	.673	1.0
model 2	1.38 (0.56–3.27)	.468	1.25 (0.64–2.40)	.504	0.96 (0.54–1.68)	.875	1.0
model 3	1.34 (0.52–3.32)	.534	1.10 (0.55–2.18)	.777	0.87 (0.48–1.55)	.631	1.0
**Visuospatial/constructional**							
model 1	4.31 (2.00–9.17)	< .01**	1.77 (0.93–3.31)	.077	1.30 (0.73–2.29)	.370	1.0
model 2	2.15 (0.87–5.22)	.092	0.85 (0.40–1.74)	.651	1.10 (0.60–2.02)	.757	1.0
model 3	2.09 (0.80–5.44)	.132	0.68 (0.31–1.46)	.334	1.02 (0.53–1.93)	.963	1.0
**Language**							
model 1	0.77 (0.28–1.85)	.592	1.46 (0.82–2.56)	.190	1.05 (0.63–1.73)	.847	1.0
model 2	0.44 (0.14–1.18)	.123	0.98 (0.50–1.86)	.943	0.94 (0.55–1.59)	.822	1.0
model 3	0.40 (0.12–1.12)	.097	0.85 (0.43–1.67)	.644	0.86 (0.49–1.48)	.586	1.0
**Attention**							
model 1	2.48 (1.05–5.53)	.031*	1.73 (0.90–3.27)	.095	2.24 (1.32–3.84)	< .01**	1.0
model 2	1.17 (0.45–2.88)	.737	0.96 (0.46–1.96)	.909	2.05 (1.18–3.60)	.012*	1.0
model 3	1.07 (0.37–2.92)	.904	0.68 (0.30–1.49)	.340	2.05 (1.12–3.82)	.021*	1.0
**Delayed memory**							
model 1	4.18 (1.76–9.61)	< .01**	1.92 (0.92–3.95)	.077	1.35 (0.70–2.64)	.369	1.0
model 2	1.68 (0.63–4.32)	.289	0.79 (0.35–1.77)	.573	1.14 (0.56–2.31)	.713	1.0
model 3	1.67 (0.61–4.45)	.312	0.70 (0.30–1.60)	.405	1.08 (0.52–2.26)	.830	1.0

Model 1: Unadjusted; model 2: Adjusted for age groups, gender, physical activity levels, diabetes, hypertension, cardiovascular disease, high cholesterol, stroke, and depression; model 3: Model 2 and years of education. OR, odds ratio; CI, confidence interval.

*p < .05,

** *p* < .01.

Table 3 presents the results on the odds of association between individual components of SO and cognitive impairment. After accounting for age groups, gender, physical activity levels, medical history and education, cognitive impairment was found to be significantly associated with low muscle strength, low physical function, and obesity. First, significant association was found between low HGS and impairment in immediate memory (OR: 1.91, 95% CI 1.04–3.49, *p* = .035). Second, low GS was also significantly associated with greater odds of impairment in the immediate memory (OR: 2.17, 95% CI 1.26–3.72, *p* < .01. Last, significant relationship was found between high FMI and impairment in attention (OR: 2.06, 95% CI 1.22–3.51, *p* < .01).

**Table 3:** Association between components of sarcopenic obesity and risk of impairment in different domains of cognitive function.

	Low ALMI (*n* = 276)		Low HGS (*n* = 99)		Low GS (*n* = 189)		High FMI (*n* = 216)	
	OR (95% CI)	*p*	OR (95% CI)	*p*	OR (95% CI)	*p*	OR (95% CI)	*p*
Global cognitive function	0.92 (0.51–1.63)	.768	1.71 (0.88–3.31)	.113	1.67 (0.91–3.03)	.096	1.48 (0.85–2.58)	.164
Immediate memory	0.87 (0.52–1.45)	.600	1.91 (1.04–3.49)	.035*	2.17 (1.26–3.72)	< .01**	0.92 (0.56–1.50)	.742
Visuospatial/constructive	0.80 (0.45–1.38)	.419	1.72 (0.91–3.23)	.094	1.40 (0.77–2.51)	.261	1.40 (0.82–2.39)	.214
Language	0.66 (0.40–1.09)	.106	0.88 (0.45–1.65)	.687	1.07 (0.61–1.83)	.811	0.79 (0.49–1.28)	.349
Attention	0.60 (0.34–1.04)	.069	1.21 (0.62–2.32)	.576	1.64 (0.91–2.92)	.095	2.06 (1.22–3.51)	< .01**
Delayed memory	0.88 (0.47–1.63)	.675	1.57 (0.81–3.02)	.180	1.65 (0.85–3.19)	.138	1.42 (0.79–2.58)	.240

Model adjusted for age groups, gender, physical activity levels, diabetes, hypertension, cardiovascular disease, high cholesterol, stroke, depression, and years of education.

ALMI, appendicular lean mass index; HGS, handgrip strength; GS, gait speed; FMI, fat mass index; OR, odds ratio; CI, confidence interval.

*p < .05,

** *p* < .01.

We performed additional analyses and found that obesity combined with low HGS or slow GS indeed showed significantly greater odds of cognitive impairment (Tables 4 and 5). After adjusting for confounding variables, co-occurrence of low HGS and high FMI was found to be associated with significantly higher odds of impairment in global cognition (OR: 2.98, 95% CI 1.08–8.19, *p* = .034), visuospatial/constructional abilities (OR: 3.70, 95% CI 1.45–9.52, *p* < .01), and attention (OR: 3.11, 95% CI 1.17–8.20, *p* = .022). Similarly, significant association was found between impairment in the attention domain and co-occurrence of slow gait speed and high FMI (OR: 3.02, 95% CI 1.41–6.51, *p* < .01).

**Table 4:** Association between handgrip strength, obesity, and risk of impairment in different domains of cognitive function.

	Low HGS and Obese (*n* = 35)		Low HGS (*n* = 64)		Obese (*n* = 181)		Normal (*n* = 255)
	OR (95% CI)	*p*	OR (95% CI)	*p*	OR (95% CI)	*p*	
Global cognitive function	2.98 (1.08–8.19)	.034*	1.58 (0.69–3.57)	.275	1.42 (0.75–2.69)	.279	1.0
Immediate memory	1.95 (0.78–4.76)	.145	1.76 (0.84–3.64)	.128	0.89 (0.50–1.57)	.695	1.0
Visuospatial/constructive	3.70 (1.45–9.52)	< .01**	1.12 (0.49–2.49)	.782	1.07 (0.57–1.99)	.837	1.0
Language	0.46 (0.14–1.31)	.166	1.08 (0.51–2.26)	.830	0.90 (0.53–1.53)	.708	1.0
Attention	3.11 (1.17–8.20)	.022*	1.03 (0.43–2.39)	.940	1.87 (1.03–3.41)	.039*	1.0
Delayed memory	2.37 (0.88–6.24)	.083	1.58 (0.67–3.66)	.284	1.44 (0.71–2.96)	.313	1.0

Model adjusted for age groups, gender, physical activity levels, diabetes, hypertension, cardiovascular disease, high cholesterol, stroke, depression, and years of education.

HGS, handgrip strength; OR, odds ratio; CI, confidence interval.

*p < .05,

** *p* < .01.

**Table 5:** Association between gait speed, obesity, and risk of impairment in different domains of cognitive function.

	Low GS and Obese (*n* = 88)		Low GS (*n* = 101)		Obese (*n* = 128)		Normal (*n* = 218)
	OR (95% CI)	*p*	OR (95% CI)	*p*	OR (95% CI)	*p*	
Global cognitive function	2.20 (0.99–4.91)	.054	1.94 (0.89–4.21)	.092	1.74 (0.81–3.73)	.151	1.0
Immediate memory	1.87 (0.92–3.78)	.083	2.29 (1.17–4.47)	.015*	0.90 (0.43–1.79)	.761	1.0
Visuospatial/constructive	1.86 (0.87–3.98)	.107	1.26 (0.59–2.65)	.547	1.26 (0.59–2.63)	.544	1.0
Language	0.86 (0.41–1.75)	.681	1.10 (0.56–2.12)	.767	0.80 (0.42–1.48)	.477	1.0
Attention	3.02 (1.41–6.51)	< .01**	1.54 (0.70–3.34)	.279	2.01 (0.98–4.11)	.056	1.0
Delayed memory	2.17 (0.93–5.10)	.073	1.59 (0.69–3.68)	.276	1.36 (0.55–3.28)	.491	1.0

Model adjusted for age groups, gender, physical activity levels, diabetes, hypertension, cardiovascular disease, high cholesterol, stroke, depression, and years of education.

GS, gait speed; OR, odds ratio; CI, confidence interval.

*p < .05,

** *p* < .01.

## Discussion

This study examined the association between SO and cognitive impairment among healthy community-dwelling adults. We found that sarcopenia with and without obesity were not associated with global or domain-specific cognitive impairment after adjusting for potential confounders. However, obesity on its own was significantly associated with greater odds of impairment in the attention domain. Furthermore, we identified that low muscle strength, low physical function and high fat mass were individually associated with impaired cognitive function.

Sarcopenia alone [6,7] and concurrent with obesity [9,21,22] have been purported to be associated with cognitive impairment. While we did observe that sarcopenia and SO were related to cognitive impairment in the unadjusted models, these associations were not significant after accounting for age, gender, and education. Increasing age and lower education levels are well established strong risk factors for Alzheimer’s disease [34], and strongly confounded the observed higher prevalence of cognitive impairment in SO and sarcopenic individuals, who were significantly much older and less educated as compared to the other groups. The diagnosis of sarcopenia requires the presence of low muscle mass as the core criterion [4]. The salient lack of independent association between low ALMI and all domains of cognitive function critically explains the insignificant association between sarcopenia and cognitive impairment.

On the other hand, we found that other functional components of sarcopenia, i.e. low HGS and GS, were individually associated with cognitive impairment even after accounting for confounding variables. These findings corroborate previous studies that suggested muscle quality is more relevant to cognitive function than muscle quantity [8,35–38]. Such results are also consistent with previous evidence of association between GS, HGS, and cognitive impairment [39,40]. Although the underlying mechanisms remain unclear, mutual pathological factors such as chronic inflammation, oxidative stress, hormonal changes, and insulin resistance have been postulated to explain the association between poor physical function and cognitive decline [41,42]. Neurological mechanisms are also plausible given that both HGS and GS performance involve multiple brain regions [43,44]. The present study findings revealed that low HGS and slow GS were associated with impairment in immediate memory domain. Future studies are warranted to elucidate the associated pathophysiological pathways.

Interestingly, we found that obesity on its own was associated with significantly greater risk of cognitive impairment. While obesity has been posited to have links with reduced cognitive function [45,46], there is criticism that the relationship between adiposity and cognitive impairment is obscured by the use of BMI to define obesity [47], since it is a measure of excess weight instead of body fat [48]. The present study is confirmatory in demonstrating that greater FMI was associated with cognitive impairment after accounting for confounding variables. Previous studies have reported poorer executive function among obese individuals [49,50]. Our results extend current body of knowledge by showing that greater adiposity was specifically associated with deficit in attention domain. This association is plausibly explained through systemic inflammation and insulin resistance [19]. Increased adipose tissues have been reported to increase secretion of proinflammatory cytokines [51] and impair insulin sensitivity [52], in which both conditions were associated with cognitive impairment among older adults [53,54].

SO was postulated to have stronger detrimental effects on cognitive function than either sarcopenia or obesity alone [9,21]. It has been demonstrated to affect multiple cognitive domains among community-dwelling adults [21] and type 2 diabetes patients [55]. In this study, we found that SO was not associated with cognitive impairment in adjusted models. This was due to the lack of independent effects of ALMI (as the core criterion of sarcopenia) on cognitive function. Our results indicate that in the absence of low ALMI, the co-occurrence of obesity with either low muscle strength or poor physical performance (as alternative definitions of sarcopenic obesity) were significantly associated with increased odds of global and domain-specific cognitive impairment. Thus, our observations strongly suggest that the impacts of sarcopenia and SO on cognitive outcome is stronger if based on muscle strength and physical performance parameters without the requisite core criterion of low ALMI.

Insulin resistance-induced endothelial dysfunction may mediate the relationship between sarcopenic obesity and cognitive function [9,56]. Beyond its catabolic effect on skeletal muscle tissues, insulin resistance has been suggested to correlate with poorer muscle function independently [57]. Thus, obese individuals with greater insulin resistance may be at greater risk of decline in muscle strength and physical performance. Since insulin resistance is not measured in present study, further research is required to examine the suggested mechanism behind the relationship between SO and cognitive impairment.

The strengths of the present study are the recruitment of a representative sample across different adult age groups and the use of a gold standard instrument to measure body composition. However, we have to acknowledge some limitations in the present study. First, as the study sample consisted of relatively healthy community-dwelling adults, caution is advised in generalising the results to individuals with more severe physical and cognitive impairments. Second, due to its cross-sectional nature, our study is not able to determine the temporality and direction of the associations identified. Future longitudinal studies are necessary to establish the specific relationship between components of SO and cognitive impairment.

In conclusion, we found no association between sarcopenic obesity based on AWGS’s definition of sarcopenia and cognitive impairment after adjusting for confounders. Low lean mass was notably not associated with cognitive impairment, but low muscle strength, low physical performance and obesity defined by high FMI were independently associated. The co-occurrence of obesity with either low muscle strength or poor physical performance was associated with exacerbated odds of observing cognitive impairment.

## Supporting information

S1 File Dataset. (CSV)

## References

[1] Patterson C. World Alzheimer report 2018: the state of the art of dementia research: new frontiers. London, UK: Alzheimer’s Disease International; 2018.

[2] Livingston G, Huntley J, Sommerlad A, Ames D, Ballard C, Banerjee S, et al. Dementia prevention, intervention, and care: 2020 report of the Lancet Commission. Lancet. 2020;396(10248):413–446. doi: 10.1016/S0140-6736(20)30367-6 32738937 PMC7392084

[3] Rosenberg IH. Sarcopenia: origins and clinical relevance. J Nutr. 1997;127(5):990S–991S. doi: 10.1093/jn/127.5.990S 9164280

[4] Chen L-K, Woo J, Assantachai P, Auyeung T-W, Chou M-Y, Iijima K, et al. Asian Working Group for Sarcopenia: 2019 consensus update on sarcopenia diagnosis and treatment. J Am Med Dir Assoc. 2020;21(3):300–307. doi: 10.1016/j.jamda.2019.12.012 32033882

[5] Beaudart C, Rizzoli R, Bruyère O, Reginster J-Y, Biver E. Sarcopenia: burden and challenges for public health. Arch Public Health. 2014;72(1):45. doi: 10.1186/2049-3258-72-45 25810912 PMC4373245

[6] Peng T-C, Chen W-L, Wu L-W, Chang Y-W, Kao T-W. Sarcopenia and cognitive impairment: A systematic review and meta-analysis. Clin Nutr. 2019;39(9):2695–2701. doi: 10.1016/j.clnu.2019.12.014 31917049

[7] Chang K-V, Hsu T-H, Wu W-T, Huang K-C, Han D-S. Association between sarcopenia and cognitive impairment: a systematic review and meta-analysis. J Am Med Dir Assoc. 2016;17(12):1164.e7–1164.e15. doi: 10.1016/j.jamda.2016.09.013 27816484

[8] Huang C-Y, Hwang A-C, Liu L-K, Lee W-J, Chen L-Y, Peng L-N, et al. Association of dynapenia, sarcopenia, and cognitive impairment among community-dwelling older Taiwanese. Rejuvenation Res. 2016;19(1):71–78. doi: 10.1089/rej.2015.1710 26165544

[9] Levine M, Crimmins E. Sarcopenic obesity and cognitive functioning: the mediating roles of insulin resistance and inflammation? Curr Gerontol Geriatr Res. 2012;2012:1–7. doi: 10.1155/2012/826398 22611388 PMC3352243

[10] Hughes VA, Roubenoff R, Wood M, Frontera WR, Evans WJ, Fiatarone Singh MA. Anthropometric assessment of 10-y changes in body composition in the elderly. Am J Clin Nutr. 2004;80(2):475–482. doi: 10.1093/ajcn/80.2.475 15277173

[11] Elias M, Elias P, Sullivan L, Wolf P, D’agostino R. Lower cognitive function in the presence of obesity and hypertension: the Framingham heart study. Int J Obes. 2003;27(2):260–268. doi: 10.1038/sj.ijo.802225 12587008

[12] Walther K, Birdsill AC, Glisky EL, Ryan L. Structural brain differences and cognitive functioning related to body mass index in older females. Hum Brain Mapp. 2010;31(7):1052–1064. doi: 10.1002/hbm.20916 19998366 PMC6870943

[13] Kuo HK, Jones RN, Milberg WP, Tennstedt S, Talbot L, Morris JN, et al. Cognitive function in normal-weight, overweight, and obese older adults: an analysis of the advanced cognitive training for independent and vital elderly cohort. J Am Geriatr Soc. 2006;54(1):97–103. doi: 10.1111/j.1532-5415.2005.00522.x 16420204 PMC2834231

[14] Noh H-M, Han J, Kim YJ, Jung J-H, Roh YK, Song HJ. Sex differences in the relationship between cognitive impairment and overweight or obesity in late life: A 3-year prospective study. Medicine. 2019;98(9):e14736. doi: 10.1097/MD.0000000000014736 30817627 PMC6831333

[15] Vidyanti AN, Hardhantyo M, Wiratama BS, Prodjohardjono A, Hu C-J. Obesity is less frequently associated with cognitive impairment in elderly individuals: A cross-sectional study in Yogyakarta, Indonesia. Nutrients. 2020;12(2):367. doi: 10.3390/nu12020367 32019161 PMC7071195

[16] Noh H-M, Oh S, Song HJ, Lee EY, Jeong J-Y, Ryu O-H, et al. Relationships between cognitive function and body composition among community-dwelling older adults: a cross-sectional study. BMC Geriatr. 2017;17(1):259. doi: 10.1186/s12877-017-0651-9 29096612 PMC5667483

[17] Waki T, Tanaka-Mizuno S, Takashima N, Takechi H, Hayakawa T, Miura K, et al. Waist Circumference and domain-specific cognitive function among non-demented Japanese older adults stratified by sex: Results from the Takashima cognition study. J Alzheimer’s Dis. 2020;73(3):887–896. doi: 10.3233/JAD-190395 31884460 PMC8590375

[18] Fitzpatrick AL, Kuller LH, Lopez OL, Diehr P, O’Meara ES, Longstreth W, et al. Midlife and late-life obesity and the risk of dementia: cardiovascular health study. Arch Neurol. 2009;66(3):336–342. doi: 10.1001/archneurol.2008.582 19273752 PMC3513375

[19] Smith E, Hay P, Campbell L, Trollor JN. A review of the association between obesity and cognitive function across the lifespan: implications for novel approaches to prevention and treatment. Obes Rev. 2011;12(9):740–755. doi: 10.1111/j.1467-789X.2011.00920.x 21991597

[20] Baumgartner RN. Body composition in healthy aging. Ann N Y Acad Sci. 2000;904(1):437–448. doi: 10.1111/j.1749-6632.2000.tb06498.x 10865787

[21] Tolea MI, Chrisphonte S, Galvin JE. Sarcopenic obesity and cognitive performance. Clin Interv Aging. 2018;13:1111–1119. doi: 10.2147/CIA.S164113 29922049 PMC5995418

[22] WangH, HaiS, LiuY, CaoL, LiuY, LiuP, et al. Associations between sarcopenic obesity and cognitive impairment in elderly Chinese community-dwelling individuals. J Nutr Health Aging. 2019;23(1):14–20. doi: 10.1007/s12603-018-1088-3 30569063

[23] KhorEQ-E, LimJ, TayL, YeoA, YewS, DingY, et al. Obesity definitions in sarcopenic obesity: Differences in prevalence, agreement and association with muscle function. J Frailty Aging. 2020;9(1):37–43. doi: 10.14283/jfa.2019.28 32150212

[24] Pang BWJ, Wee SL, Lau LK, Jabbar KA, Seah WT, Ng DHM, et al. Obesity Measures and Definitions of Sarcopenic Obesity in Singaporean Adults–the Yishun Study. J Frailty Aging. Forthcoming 2020. doi: 10.14283/jfa.2020.65 34105702

[25] Pang BWJ, Wee SL, Lau LK, Jabbar KA, Seah WT, Ng DHM, et al. Prevalence and associated factors of sarcopenia in Singaporean adults–the Yishun Study. J Am Med Dir Assoc. Forthcoming 2020. doi: 10.1016/j.jamda.2020.05.029 32693999

[26] Kim TN, Yang S, Yoo H-J, Lim K, Kang H, Song W, et al. Prevalence of sarcopenia and sarcopenic obesity in Korean adults: the Korean sarcopenic obesity study. Int J Obes. 2009;33(8):885–892. doi: 10.1038/ijo.2009.130 19564878

[27] Peltz G, Aguirre MT, Sanderson M, Fadden MK. The role of fat mass index in determining obesity. Am J Hum Biol. 2010;22(5):639–647. doi: 10.1002/ajhb.21056 20737611 PMC2929934

[28] Rantanen T, Guralnik JM, Foley D, Masaki K, Leveille S, Curb JD, et al. Midlife hand grip strength as a predictor of old age disability. JAMA. 1999;281(6):558–560. doi: 10.1001/jama.281.6.558 10022113

[29] Studenski S, Perera S, Patel K, Rosano C, Faulkner K, Inzitari M, et al. Gait speed and survival in older adults. JAMA. 2011;305(1):50–8. doi: 10.1001/jama.2010.1923 21205966 PMC3080184

[30] Kalinkovich A, Livshits G. Sarcopenic obesity or obese sarcopenia: a cross talk between age-associated adipose tissue and skeletal muscle inflammation as a main mechanism of the pathogenesis. Ageing Res Rev. 2017;35:200–221. doi: 10.1016/j.arr.2016.09.008 27702700

[31] Randolph C, Tierney MC, Mohr E, Chase TN. The Repeatable Battery for the Assessment of Neuropsychological Status (RBANS): preliminary clinical validity. J Clin Exp Neuropsychol. 1998;20(3):310–319. doi: 10.1076/jcen.20.3.310.823 9845158

[32] Randolph C. Repeatable Battery for the Assessment of Neuropsychological Status manual. San Antonio, TX: Psychological Corporation; 1998.

[33] Armstrong T, Bull F. Development of the world health organization global physical activity questionnaire (GPAQ). J Public Health. 2006;14(2):66–70. doi: 10.1007/s10389-006-0024-x

[34] Lindsay J, Laurin D, Verreault R, Hébert R, Helliwell B, Hill GB, et al. Risk factors for Alzheimer’s disease: a prospective analysis from the Canadian Study of Health and Aging. Am J Epidemiol. 2002;156(5):445–453. doi: 10.1093/aje/kwf074 12196314

[35] Abellan van Kan G, Cesari M, Gillette-Guyonnet S, Dupuy C, Nourhashémi F, Schott A-M, et al. Sarcopenia and cognitive impairment in elderly women: results from the EPIDOS cohort. Age Ageing. 2013;42(2):196–202. doi: 10.1093/ageing/afs173 23221099

[36] Auyeung TW, Kwok T, Lee J, Leung PC, Leung J, Woo J. Functional decline in cognitive impairment–the relationship between physical and cognitive function. Neuroepidemiology. 2008;31(3):167–173. doi: 10.1159/000154929 18784415 PMC2824577

[37] AuyeungTW, LeeJ, KwokT, WooJ. Physical frailty predicts future cognitive decline—a four-year prospective study in 2737 cognitively normal older adults. J Nutr Health Aging. 2011;15(8):690–694. doi: 10.1007/s12603-011-0110-9 21968866

[38] Sui SX, Holloway-Kew KL, Hyde NK, Williams LJ, Leach S, Pasco JA. Muscle strength and gait speed rather than lean mass are better indicators for poor cognitive function in older men. Sci Rep. 2020;10, 10367. doi: 10.1038/s41598-020-67251-8 32587294 PMC7316855

[39] Beauchet O, Annweiler C, Callisaya ML, De Cock A-M, Helbostad JL, Kressig RW, et al. Poor gait performance and prediction of dementia: results from a meta-analysis. J Am Med Dir Assoc. 2016;17(6):482–490. doi: 10.1016/j.jamda.2015.12.092 26852960 PMC5319598

[40] Kobayashi-Cuya KE, Sakurai R, Suzuki H, Ogawa S, Takebayashi T, Fujiwara Y. Observational evidence of the association between handgrip strength, hand dexterity, and cognitive performance in community-dwelling older adults: a systematic review. J Epidemiol. 2018;28(9):373–381. doi: 10.2188/jea.JE20170041 29526916 PMC6111109

[41] Robertson DA, Savva GM, Kenny RA. Frailty and cognitive impairment—a review of the evidence and causal mechanisms. Ageing Res Rev. 2013;12(4):840–851. doi: 10.1016/j.arr.2013.06.004 23831959

[42] Chou M-Y, Nishita Y, Nakagawa T, Tange C, Tomida M, Shimokata H, et al. Role of gait speed and grip strength in predicting 10-year cognitive decline among community-dwelling older people. BMC Geriatr. 2019;19,186. doi: 10.1186/s12877-019-1199-7 31277579 PMC6612180

[43] Carson RG. Get a grip: individual variations in grip strength are a marker of brain health. Neurobiol Aging. 2018;71:189–222. doi: 10.1016/j.neurobiolaging.2018.07.023 30172220

[44] Harada T, Miyai I, Suzuki M, Kubota K. Gait capacity affects cortical activation patterns related to speed control in the elderly. Exp Brain Res. 2009;193(3):445–454. doi: 10.1007/s00221-008-1643-y 19030850

[45] Norton S, Matthews FE, Barnes DE, Yaffe K, Brayne C. Potential for primary prevention of Alzheimer’s disease: an analysis of population-based data. Lancet Neurol. 2014;13(8):788–794. doi: 10.1016/S1474-4422(14)70136-X 25030513

[46] Prickett C, Brennan L, Stolwyk R. Examining the relationship between obesity and cognitive function: a systematic literature review. Obes Res Clin Pract. 2015;9(2):93–113. doi: 10.1016/j.orcp.2014.05.001 25890426

[47] Papachristou E, Ramsay SE, Lennon LT, Papacosta O, Iliffe S, Whincup PH, et al. The relationships between body composition characteristics and cognitive functioning in a population-based sample of older British men. BMC Geriatr. 2015;15,172. doi: 10.1186/s12877-015-0169-y 26692280 PMC4687114

[48] Prentice AM, Jebb SA. Beyond body mass index. Obes Rev. 2001;2(3):141–147. doi: 10.1046/j.1467-789x.2001.00031.x 12120099

[49] Gunstad J, Paul RH, Cohen RA, Tate DF, Spitznagel MB, Gordon E. Elevated body mass index is associated with executive dysfunction in otherwise healthy adults. Compr Psychiatry. 2007;48(1):57–61. doi: 10.1016/j.comppsych.2006.05.001 17145283

[50] Sabia S, Kivimaki M, Shipley MJ, Marmot MG, Singh-Manoux A. Body mass index over the adult life course and cognition in late midlife: the Whitehall II Cohort Study. Am J Clin Nutr. 2009;89(2):601–607. doi: 10.3945/ajcn.2008.26482 19073790 PMC2714395

[51] Balistreri CR, Caruso C, Candore G. The role of adipose tissue and adipokines in obesity-related inflammatory diseases. Mediators Inflamm. 2010;2010,802078. doi: 10.1155/2010/802078 20671929 PMC2910551

[52] Qatanani M, Lazar MA. Mechanisms of obesity-associated insulin resistance: many choices on the menu. Genes Dev. 2007;21(12):1443–1455. doi: 10.1101/gad.1550907 17575046

[53] Trollor JN, Smith E, Baune BT, Kochan NA, Campbell L, Samaras K, et al. Systemic inflammation is associated with MCI and its subtypes: the Sydney Memory and Aging Study. Dement Geriatr Cogn Disord. 2010;30(6):569–578. doi: 10.1159/000322092 21252552

[54] Abbatecola AM, Paolisso G, Lamponi M, Bandinelli S, Lauretani F, Launer L, et al. Insulin resistance and executive dysfunction in older persons. J Am Geriatr Soc. 2004;52(10):1713–1718. doi: 10.1111/j.1532-5415.2004.52466.x 15450050

[55] Low S, Goh KS, Ng TP, Ang SF, Moh A, Wang J, et al. The prevalence of sarcopenic obesity and its association with cognitive performance in type 2 diabetes in Singapore. Clin Nutr. 2020;39(7):2274–2281. doi: 10.1016/j.clnu.2019.10.019 31744622

[56] Buie JJ, Watson LS, Smith CJ, Sims-Robinson C. Obesity-related cognitive impairment: The role of endothelial dysfunction. Neurobiol Dis. 2019;132,104580. doi: 10.1016/j.nbd.2019.104580 31454547 PMC6834913

[57] Stenholm S, Harris TB, Rantanen T, Visser M, Kritchevsky SB, Ferrucci L. Sarcopenic obesity-definition, etiology and consequences. Curr Opin Clin Nutr Metab Care. 2008;11(6):693–700. doi: 10.1097/MCO.0b013e328312c37d 18827572 PMC2633408

